# Modelling Renal Filtration and Reabsorption Processes in a Human Glomerulus and Proximal Tubule Microphysiological System

**DOI:** 10.3390/mi12080983

**Published:** 2021-08-19

**Authors:** Stephanie Y. Zhang, Gretchen J. Mahler

**Affiliations:** Department of Biomedical Engineering, The State University of New York at Binghamton, Binghamton, NY 13902, USA; szhan152@binghamton.edu

**Keywords:** kidney, cytoskeleton, microfluidics, cytotoxicity, pharmacokinetics

## Abstract

Kidney microphysiological systems (MPS) serve as potentially valuable preclinical instruments in probing mechanisms of renal clearance and osmoregulation. Current kidney MPS models target regions of the nephron, such as the glomerulus and proximal tubule (PCT), but fail to incorporate multiple filtration and absorption interfaces. Here, we describe a novel, partially open glomerulus and PCT microdevice that integrates filtration and absorption in a single MPS. The system equalizes pressure on each side of the PCT that operates with one side “closed” by recirculating into the bloodstream, and the other “opened” by exiting as primary filtrate. This design precisely controls the internal fluid dynamics and prevents loss of all fluid to the open side. Through this feature, an in vitro human glomerulus and proximal tubule MPS was constructed to filter human serum albumin and reabsorb glucose for seven days of operation. For proof-of-concept experiments, three human-derived cell types—conditionally immortalized human podocytes (CIHP-1), human umbilical vein endothelial cells (HUVECs), and human proximal tubule cells (HK-2)—were adapted into a common serum-free medium prior to being seeded into the three-component MPS (T-junction splitter, glomerular housing unit, and parallel proximal tubule barrier model). This system was optimized geometrically (tubing length, tubing internal diameter, and inlet flow rate) using in silico computational modeling. The prototype tri-culture MPS successfully filtered blood serum protein and generated albumin filtration in a physiologically realistic manner, while the device cultured only with proximal tubule cells did not. This glomerulus and proximal convoluted tubule MPS is a potential prototype for the human kidney used in both human-relevant testing and examining pharmacokinetic interactions.

## 1. Introduction

Biopharmaceutical research and development faces a major productivity crisis in the depreciating efforts to develop novel drugs [1]. Despite over 30 years of investment in biomedical sciences and the scientific tools used in drug discovery, few results have been well translated in the preclinical and clinical stages [2,3]. The reliance on conventional cell culture systems and animal models during preclinical testing hinders the establishment of human-related kidney predictive models [4,5]. Microphysiological systems (MPS) can accurately model human systems in a compact, efficient fluidic tool that can introduce controlled spatiotemporal micro-environments [6]. Such systems support measuring human responses, allow for real-time imaging, encourage cell differentiation, and pinpoint cell–cell interactions under a variety of physiological conditions [7,8,9,10,11]. Recent developments in stem cell research [12,13], regenerative medicine [14], biomaterials [15,16], tissue engineering [17,18,19,20], and microfluidics allow for integration into three-dimensional (3D) MPS.

**Figure 1:**
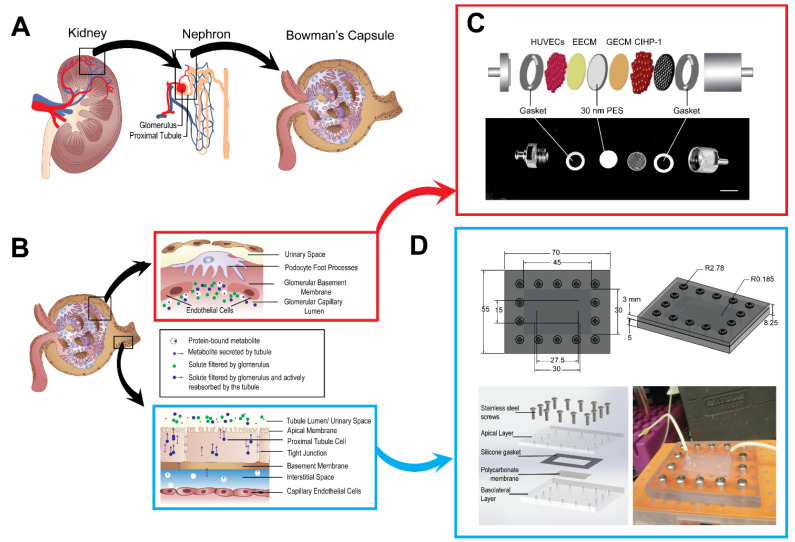
Translation of the nephron into MPS. (**A**,**B**) Schematic of kidney, nephron, and its structures. (**C**) Schematic and image of glomerulus housing unit. Bar = 12 mm. (**D**) Schematic and image of PCT device.

In the kidney, millions of nephrons employ filtration, reabsorption, secretion, and excretion processes. Each functional unit collaborates to filter out wastes and xenobiotics; separate water, ions, and small molecules from the blood; and recycle compounds to the blood (Figure 1A,B) [21]. Within the nephron, glomerular filtration consists of passive movement of plasma from glomerulus capillaries to the Bowman’s capsule that is freely permeable to water and small solutes (Na^+^, urea, and glucose), but not permeable to blood, white blood cells, platelets, or large-molecular-weight (>67 kDa) serum proteins (albumin) [22]. The glomerular filtrate exits the glomerulus to enter a selective barrier of highly coiled tubules. There, the proximal convoluted tubule (PCT) utilizes active and passive transport to reabsorb glucose, sodium chloride, and water from the glomerular filtrate [21,23]. During reabsorption, highly concentrated filtrate becomes the leading site for nephrotoxin accumulation, a precursor for acute kidney injury or chronic kidney disease. Nearly 90% of renal toxicity cases are derived from both the glomerulus and PCT of the kidney [8,20,24]. Current MPS designs of the kidney have been well established for the PCT segment of the nephron, representing only a section of the renal absorption process [7,8,11,20,25,26]. However, incorporating both filtration and absorption interfaces will refine the physiological relevance of the kidney in vitro barrier model, and permit rapid screening for drug toxicity in preclinical studies.

In this study, a novel MPS of the proximal tubule and glomerulus that highlights crossflow filtration and is capable of long-term operation has been developed. Here, we describe a novel microscale system to incorporate filtration and potentially reabsorption and secretion. They are designed to equalize pressure on each side for the PCT that allows operation with one side “closed” and the other “opened.” This design allows control of the fluid dynamics and prevents the loss of all the fluid to the open side. This feature allows construction of an in vitro MPS that emulates key filtration of serum albumin and glucose reabsorption properties in the human glomerulus and PCT for seven days of operation.

As a test of this prototype system, we used a tri-culture system incorporating conditionally immortalized human podocytes (CIHP-1) to represent the ultrafiltration processes from the fenestrations in the glomerular capsule, human umbilical vein endothelial cells (HUVECs) for the capillaries recirculating solutes in the bloodstream, and human kidney-2 (HK-2) to recreate the reabsorption processes in the PCT. Key design requirements included integration of the glomerular filtration fraction (0.15–0.2) and tubular reabsorption (0.65–0.7) [21,27]. The cells within the proximal tubule were grown under dynamic flow engineered to mimic flow conditions in vivo (0.4–1.2 dyne-s/cm^2^) [4] for seven days, and then the system was challenged with fluorescein isothiocyanate-human serum albumin (FITC-HSA) to assess its filtration functional capacity. MPS culture medium determination and imaging were completed without the introduction of animal by-products. We show here that the prototype tri-culture MPS successfully filtered blood serum protein, resorbed glucose, and generated albumin filtration in a physiologically realistic manner. This MPS introduces a novel physical model of a PCT and glomerulus with the capability of blood serum protein filtration, glucose resorption, and filtrate formation.

## 2. Materials and Methods

### 2.1. Simulating and Validating Model in COMSOL MultiPhysics^®^

A 2D computational model was developed through COMSOL MultiPhysics (COMSOL Inc., Burlington, MA, USA). Computational domains were generated using COMSOL’s native geometry tools. To model the fluid flow in open and porous domains, the Free and Porous Media Flow physics package was used for porous membranes. Inlet, Outlet and No-Slip Wall conditions were used to define the boundary conditions of the computational domain. A symmetry boundary condition was used to reduce the computational load, and domains were discretized using a combination of swept, tetrahedral and boundary layer elements using native meshing tools. Using both stationary and time-dependent solvers, the 2D model was used to optimize tubing internal diameter (ID) and length (Cole-Parmer, Vernon Hills, IL, USA), and total flow rate from the peristaltic pump (Watson-Marlow, Paramus, NJ, USA). The primary design parameters achieved a passive fluid flow split at the glomerular filter T-junction (Eldon James, Denver, CO, USA), and a fluid shear stress of 0.65 dyn-s/cm^2^ across the top chamber of the proximal tubule device (PCT), which mimics the physiological fluid shear stress in the human proximal tubule.

### 2.2. Fabrication of the Microphysiological System

Construction of the proximal tubule-on-a-chip design has been previously described [11,28]. Briefly, the CNC-milled, polycarbonate device contains two fluidic channels (30 × 15 mm filtrate channel and 30 × 15 mm bloodstream channel) with a total height of 50 µm, and two inlet and outlet ports for all fluidic channels. The filtrate and bloodstream channels were separated by a porous polycarbonate membrane (porosity = 20, pore size = 0.4 µm). The porous polycarbonate membrane was sealed with a 0.01 in thick silicone polymer gasket (McMaster-Carr, Elmhurst, IL, USA) and 14-4-40 5/16 in stainless steel screws (APM HEXSEAL), using an autoclavable stainless steel screwdriver with Nylon Handle 4″ (100 mm) OAL (Philips, Amsterdam, The Netherlands).

### 2.3. Cell Culture

All cell lines were adapted to endothelial serum-free medium (ESFM, Thermo Fisher Scientific, Waltham, MA, USA) supplemented with 10 µg/mL of human plasma fibronectin (Thermo Fisher Scientific, Waltham, MA, USA), 20 ng/mL of human recombinant epidermal growth factor (EGF, Thermo Fisher Scientific, Waltham, MA, USA), and 10 ng/mL of human recombinant basic fibroblast growth factor (bFGF, Thermo Fisher Scientific, Waltham, MA, USA). Conditionally immortalized human podocytes (CIHP-1) purchased from Dr. Saleem Moin’s laboratory at the University of Bristol at passage 16 proliferated at 33 °C in 5% CO_2_ and were passaged every 10–14 days. The human, renal proximal tubule cell line (HK-2) (passage 20–30) obtained from the American Type Culture Collection (ATCC, Manassas, VA) was incubated at 37 °C in 5% CO_2_ and passaged every 5–7 days with 0.05% trypsin-EDTA (Thermo Fisher Scientific, Waltham, MA, USA). Human umbilical vein endothelial cells (HUVECs) purchased from Lonza Bioscience (Rockland, MD, USA) were maintained at 37 °C in 5% CO_2_ and experiments were completed using passages 3–7.

### 2.4. Cell Viability and Proliferation Assay

To optimize the proliferation and viability of all three cell types, a growth curve was constructed with four types of media: Dulbecco’s Modified Eagle medium supplemented with 10% fetal bovine serum (FBS, Thermo Fisher Scientific, Waltham, MA, USA), kerotinocyte serum-free medium (KSFM) supplemented with 1% insulin-transferrin-selenium (ITS, Thermo Fisher Scientific, Waltham, MA, USA), ESFM (Thermo Fisher Scientific, Waltham, MA, USA), and Roswell Park Memorial Institute medium (RPMI)-1640 supplemented with 10% FBS and 1% ITS (Thermo Fisher Scientific, Waltham, MA, USA). In a 24-well plate (Corning, Corning, NY, USA), each cell type was seeded at 40,000 cells/cm^2^ (*n* = 60) and independently grown in each medium type at their respective temperatures (33 °C or 37 °C) with 5% CO_2_ for 96 h. Every 24 h, cells were either replenished with new medium or removed for cell counting. Wells (*n* = 3) for each cell and medium type were separately trypsinized, stained with Trypan Blue, and counted using a hemocytometer. In addition, HK-2, CIHP-1, and HUVECs were grown in static condition on their respective membranes for 7 days and stained with phallodin 568 and Hoescht 33342 to confirm viability and attachment.

### 2.5. CIHP-1, HUVECs, and HK-2 Adhesion on Porous Membranes

The 30 nm pore size polyethersulfone (PES) membranes (Sterlitech, Kent, WA, USA) were coated with glomerular extracellular matrix (GECM), which consist of a tri-layer of 8 µg/cm^2^ rat-tail collagen 1 (BD Biosciences, Franklin Lakes, NJ, USA), 8 µg/cm^2^ heparin sulfate (Sigma-Aldrich H7640), and 8 µg/cm^2^ rat-tail collagen 1 (BD Biosciences, Franklin Lakes, NJ, USA). The selection of the three-layer GECM composition was adapted from previous work [11,28]. For the first layer of GECM, rat-tail collagen 1 at 8 µg/cm^2^ was surface coated on the PES membrane for one hour at room temperature and washed with 1X phosphate-buffered saline (PBS) (Thermo Fisher Scientific, Waltham, MA, USA). For the second layer, heparin sulfate at 8 µg/cm^2^, responsible for cell surface binding [29], was then coated on the PES membrane to generate for another hour and washed with 1X PBS. For the third layer, another 8 µg/cm^2^ of rat-tail collagen 1 was applied and washed with 1X PBS. The three-layer coating yielded the highest podocyte attachment in comparison to single ECM coatings. The PES membrane was held in place with a custom fabricated polycarbonate holder that fits inside of a 6-well plate (Corning, Corning, NY, USA). CIHP-1 cells are seeded at a density of 100,000 cells/ cm^2^ onto the PES membranes, thermo-switched to 37 °C, and differentiated for 14 days. Differentiation is completed when cells demonstrate a morphological change on day 14. On day 12, the PES membranes holding CIHP-1 were flipped and HUVECs were seeded at 100,000 cells/cm^2^ on the other side of the membrane coated with endothelial extracellular matrix (EECM). EECM consisted of a coating of 8 µg/cm^2^ human fibronectin (Corning, Corning, NY, USA) for 1 h at room temperature. Similarly, HK-2 cells were seeded onto fibronectin-coated (8 μg/cm^2^), 0.4 µm pore size polycarbonate membrane (Whatman) at a cell density of 100,000 cells/cm^2^ for 24 h before full device assembly.

### 2.6. Assembly of the Housing Unit and the Microfluidic Device

The experimental study was conducted in a sterile, biosafety cabinet cleaned with 70% ethanol. All device components were autoclaved prior to experimentation. The internal and external components of the peristaltic pump were cleaned with 70% ethanol before stringing the Pharmed BPT 0.25 mm ID tubing (Cole-Parmer, Vernon Hills, IL, USA) into the cassette with a conical tube of 1X phosphate-buffered saline (PBS). All tubing was subjected to an average flow of 40 µL/min with 1X PBS for 1 h to eradicate bubble formation. Assembly of the glomerulus stainless steel housing unit (Advantec MFS, Dublin, CA, USA) [8,11,28], consisting of a stainless steel mesh (Advantec MFS, Dublin, CA, USA), pre-cut 14 mm in diameter PES membrane (Sterlitech, Kent, WA, USA), polytetrafluoroethylene (PTFE) gasket (Advantec MFS, Dublin, CA, USA), pre-cut 200 µL pipet tips (VWR International, Radnor, PA, USA), and Leur locks (Cole-Parmer, Vernon Hills, IL, USA), was sealed with an autoclavable wrench (SteriTool, Jacksonville, FL, USA). While the assembled glomerulus housing unit was attached to the end of the tubing to fill with 1X PBS, assembly of the T-junction and the CNC machined, polycarbonate PCT device was completed. Within the PCT device, a 0.4 µm pore size polycarbonate membrane and 0.01 in thick silicone polymer gasket (McMaster-Carr, Elmhurst, IL, USA) created an in vitro barrier system with an apical and basolateral sections. The components are assembled in their respective locations after filling with 1X PBS, and placeholder porous membranes were replaced with the cell seeded porous membranes. Afterwards, the inlet fluid was replaced with the supplemented ESFM incubated at 37 °C, and the two outlet tubing were connected to a 50 mL conical tube for filtrate output and bloodstream output. ESFM was changed every 2–3 days for a week.

### 2.7. MPS Cellular Models

Under dynamic conditions, two cellular models (single and tri-cultures) were employed with the glomerulus and PCT MPS components to assess the necessity of a multi-culture model. The single culture contained only proximal tubule cells (HK-2) adhered to the polycarbonate membrane in the PCT device. The single-culture MPS was operated with the T-junction, the stainless steel glomerulus housing unit with a blank PES membrane, and seeded HK-2 cells in the PCT device. There were no glomerular or endothelial cells (CIHP-1 and HUVECs) on the PES membranes. The tri-culture housed all three cell types on their respective membranes. The podocytes (CIHP-1) and endothelial (HUVECs) cells were attached to the PES membrane in the glomerulus unit in addition to the proximal tubule (HK-2) cells attached to the polycarbonate membrane in the PCT device.

### 2.8. Volume Flow Assay

Filtrate output volume was collected daily in 50 mL conical tubes (Corning, Corning, NY, USA) and stored in −20 °C for future assessment. In post-experiment testing, the volumes were thawed, weighed, and normalized to an empty conical tube. Based on the density of the volume (1 g/mL), total volume was calculated over time in mL/min.

### 2.9. FITC-Human Serum Albumin Flow Assay

After seven days of fluidic culture, the MPS was subjected to 0.1 mg/mL of FITC-HSA (Abcam) diluted with 1X PBS by replacing the inlet conical tube fluid. In two black 96-well plates, samples (*n* = 4) of the inlet and outlet fluid flow were collected at 15 min intervals for 2 h. Fluorescence was assessed with Synergy Plate Reader (Biotek, Winooski, VT, USA) and Gen5 software with an emission/excitation wavelength of 485/528 nm. Results were normalized to the average maximum fluorescent intensity.

### 2.10. Fluorescent Staining and Image Processing

Following system challenge with HSA, membranes were removed from the devices and placed in a 4-well plate. The cells were fixed with 4% paraformaldehyde (PFA) diluted in 1X PBS for 5 min at room temperature and permeabilized with 0.1% Triton X-100 in 1X PBS for 15 min. The cells were stained with phallodin 568 (Invitrogen) and counterstained with Hoescht 33342 (Thermo Fisher Scientific, Waltham, MA, USA) for 30 min. Membranes were then washed three times with 1X PBS for each 5 min. Samples were mounted onto 24 × 40 × 0.13 mm^3^ glass slides through ProLong Gold and visualized with Zeiss Leica SP5X confocal microscope. Images were processed in FIJI ImageJ [30] for directionality. Z-stacks were taken for the membranes to generate a three-dimensional (3D) profile.

### 2.11. Infinity Glucose Assay

Glucose concentrations were determined from the filtrate output volume using the Infinity™ Glucose Hexokinase Liquid Stable Reagent assay (Thermo Scientific, Waltham, MA, USA). The reagent was diluted with filtrate output volume at a ratio of 150:1, incubated in 37 °C for three minutes, and plated into a 96-well plate (*n* = 4) for each day. Absorbance was assessed with Synergy Plate Reader (Biotek, Winooski, VT, USA) and Gen5 software with a primary/secondary wavelength of 340/380 nm. Standards were established with both a glucose solution of 1 mg/mL of 0.1% benzoic acid and supplemented ESFM.

### 2.12. Statistical Analysis

Non-parametric one-way ANOVA with post hoc Dunn’s test, the Mann–Whitney test, and regression tests were conducted in GraphPad Prism^®^ (GraphPad, San Diego, CA, USA). All data from single- and tri-cultures were presented with medians and ranges.

## 3. Results

### 3.1. A Glomerular and Proximal Tubular Microphysiological System

**Figure 2:**
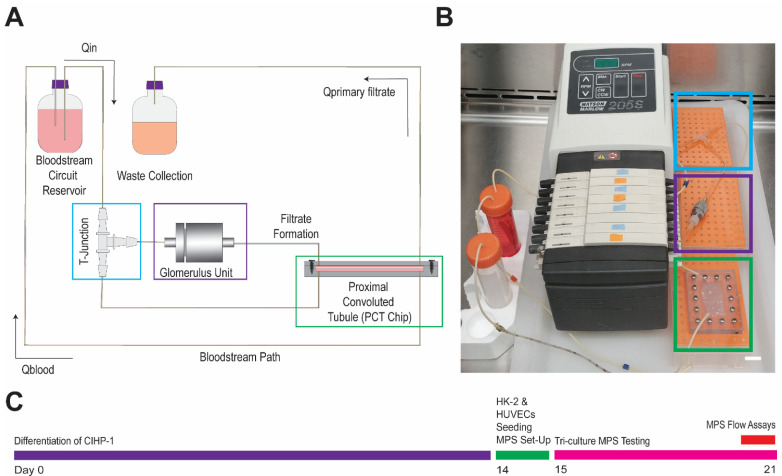
Construction of glomerulus and proximal convoluted tubule (PCT) MPS. (**A**) Schematic of MPS. (**B**) Image of MPS set-up. Bar = 15 mm. Blue = T-junction. Purple = glomerulus housing unit. Green = PCT device. (**C**) Timeline of MPS experiment.

Filtration and reabsorption of the glomerulus and PCT were assessed by two main devices in the construction of the MPS (Figure 1C,D). Within the stainless steel housing unit (Figure 1C), the glomerulus enclosed a single porous PES membrane (porosity = 60) seeded with the differentiated CIHP-1 and HUVECs. The differentiated podocytes were cultured onto the GECM- and EECM-coated PES membranes 14 days prior to initiation of flow. The negatively charged GECM and EECM generates a charge-selective filtration barrier of the glomerulus [8]. During differentiation, podocytes were observed for flattened morphological characteristics with finger-like projections in areas surrounding PES membranes. On day 15, PES membranes were flipped and seeded with the endothelial cells for 4 h. Once the HK-2 cells were seeded onto a fibronectin-coated polycarbonate membrane (porosity = 20) for 4 h, it was sealed in the machined bi-layer, polycarbonate microfluidic device described by previous work (Figure 1D) [11,28]. Its physical dimensions (30 mm long × 15 mm wide × 50 μm thick) are comparatively similar to reported in vivo PCT dimensions in the human kidney (14 mm long × 40 μm thick) [31]. The fully constructed MPS was connected to a T-junction (Figure 2A,B), a splitter of fluid flow to recycle 90% of flow into the bloodstream and 10% into the filtrate output. While the peristaltic pump maintained the MPS flow rate for the seven-day operation, additional modeling was required to optimize tubing length, diameter, and to safeguard physiological flow rates and shear rates (0.4–1.5 dynes/cm^2^) within both the glomerular housing unit and the PCT microfluidic device.

### 3.2. Flow Characterization of Microphysiological System

**Figure 3:**
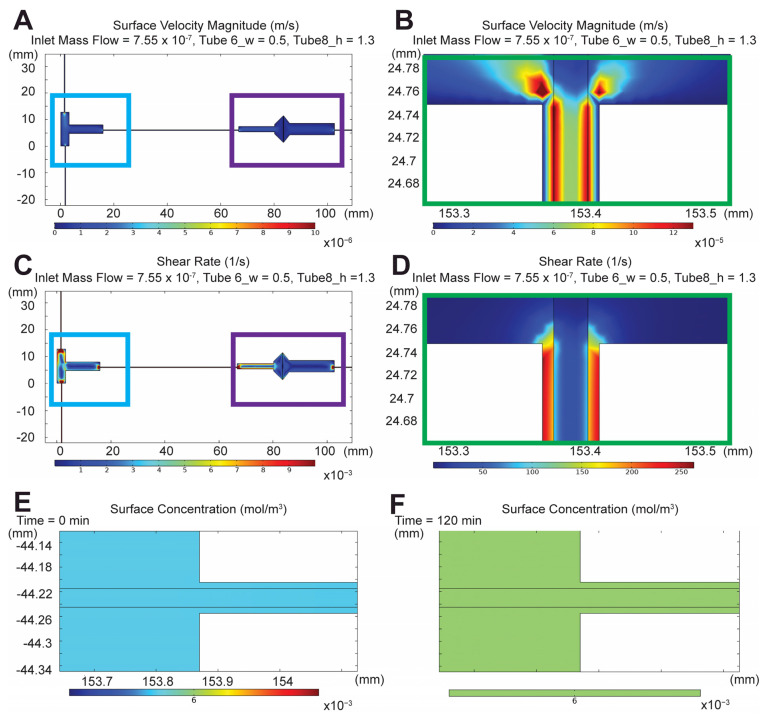
2D COMSOL^®^ velocity magnitude, shear rates, and concentration profiles. (**A**) Velocity magnitude (m/s) in T-junction and glomerular housing unit. (**B**) Velocity magnitude (m/s) in proximal convoluted tubule (PCT) device. (**C**) Shear tate (S^−1^) in T-junction and glomerular housing unit. (**D**) Shear rate (S^−1^) in PCT device. (**E**) Protein concentration (mg/mL) at time 0 min. (**F**) Protein concentration (mg/mL) at time 120 min. X and Y axes = XY geometry coordinates (mm). Blue = T-junction. Purple = glomerulus housing unit. Green = PCT device. Color bar ranges from lowest (blue) to highest measurements (red), where (**A**) 0–10 × 10^−5^ m/s, (**B**) 0–12 × 10^−5^ m/s, (**C**) 0–12 × 10^−3^ S^−1^, (**D**) 0–250 S^−1^, (**E**) 0–6 × 10^−3^ mol/m^3^, and (**F**) 0–6 × 10^−3^ mol/m^3^.

To mediate the MPS microenvironment, a two-dimensional (2D) numerical simulation of porous media transport was performed using a finite element model of the device via COMSOL MultiPhysics^®^. A series of parametrizations (Table 1) was performed for the velocity flow (m/s) (Figure 3A,B) and shear rate (1/S) (Figure 3C,D) based on the assumptions of steady state, constant fluid flow, viscosity and density, incompressible flow, minimal fluid evaporation, and negligible gravity. Conversions into physiological measurements were adopted using the following shear stress (Equation (1)).

(1) $$\tau =\frac{6Q\mu }{bh^{2}}$$

where *μ* is the endothelial serum-free culture medium viscosity at 37  °C; τ is the shear stress (dyn-s/cm^2^); *Q* is the volumetric flow rate (cm^3^/s); *b* is the channel width; and *h* is the channel height (*Q* = 7.6 × 10^−4^ cm^3^/s, *μ* = 8.9 × 10^−3^ dyn-s/cm^2^, *h* = 0.05 mm, *b* = 30 mm).

Based on the conversions, the optimal tubing length of filtrate output and bloodstream were ~508 mm and 734 mm, respectively. The tubing length ranges were selected based on peristaltic pump and incubator sizing constraints. The inlet mass flow rate was 7.6 × 10^−7^ kg/s (7.6 × 10^−4^ cm^3^/s) to generate a shear stress of 0.5 dyne-s/cm^2^ across the apical layer of the PCT device. Means of 1-D velocity profiles from filtrate output and the bloodstream had a ratio of 83/81 (Figure 3). Concentration profiles of 0.1 mg/mL FITC-HSA were computed using its diffusivity coefficient (61 μm^2^/s) in transport by diluted species with a time-dependent solver from 0 to 120 min (Figure 3E,F). The concentration 1-D profile was characterized with an exponential increase until it plateaued and approached steady state at approximately 105–120 min. While these simulations are effective in determining the optimal geometry for the desired shear stress, the model was idealistic and reproduced without the inclusion of cellular effects that may alter the overall outcome.

**Figure 4:**
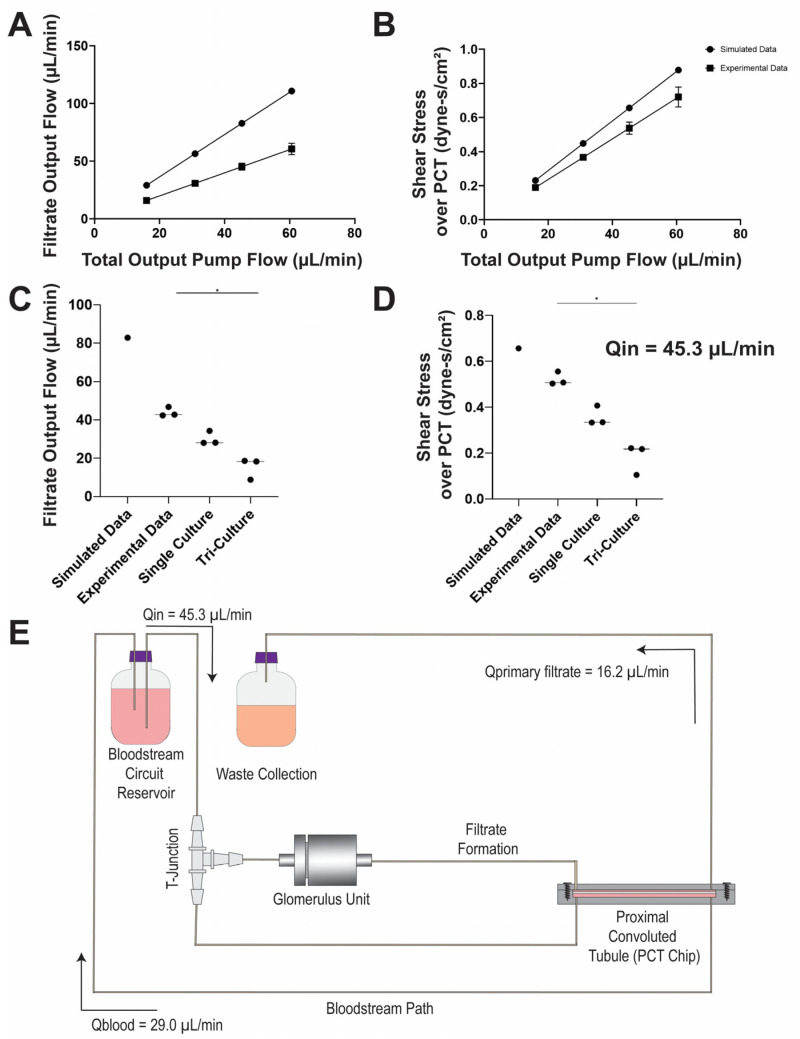
Simulated and experimental comparison of glomerulus and proximal convoluted tubule (PCT) MPS pump flow and shear stress. (**A**) Measured filtrate output flow (μL/min) for different total output pump flow (μL/min) in both simulated and experimental settings (0.25 mm ID tubing only). R^2^ = 1.0. (**B**) Shear stress across the apical PCT (dyne-s/cm^2^) for different total output pump flow (μL/min) in both simulated and experimental settings (0.25 mm ID tubing only). R^2^ = 1.0. (**C**) Scatter plot of simulated, experimental (single inlet tubing), single-culture, and tri-culture filtrate output flow under Q_in_ = 45.3 μL/min. (**D**) Scatter plot of simulated, experimental (single inlet tubing), single-culture, and tri-culture shear stress (dyne-s/cm^2^) under Q_in_ = 45.3 μL/min. (**E**) Assembled MPS flow validation. Line represents median. (* Comparison of datapoints within conditional test, *p* < 0.05, non-parametric one-way ANOVA.)

Inlet mass flow rate (kg/s) was experimentally validated by using a single inlet 0.25 ID mm tubing at a 4–16 rpm pump rate (0.19–0.72 dyne-s/cm^2^) (Figure 4A,B). Flow rate (μL/min) and shear stress (dyne-s/cm^2^) were determined with pumping 1X phosphate-buffered saline (PBS) over the course of 2 h, and collecting samples at 15 min intervals (*n* = 4 for each rpm). At a 12 rpm pump rate, the flow inlet (Q_in_) achieved an average of 45.3 ± 3.0 μL/min with a shear rate of 0.5 ± 0.04 dyne-s/cm^2^. With the assembled MPS, 1X PBS was cyclically pumped for the same duration, and filtrate formation (Q_primary filtrate_) and bloodstream recycling (Q_blood_) rates were an average of 16.2 ± 4.5 μL/min and 29.0 ± 4.5 μL/min, respectively. Filtrate output and shear stress across the PCT for the single- and tri-culture conditions (Figure 4C,D) were compared with the simulated and experimental settings to characterize the system under non-ideal conditions. Single-culture devices contained only the proximal tubule (HK-2) cells seeded into the PCT device, while the tri-culture was seeded with CIHP-1 and HUVECs in the glomerulus housing unit along with the HK-2 cells in the PCT. Implementation of single culture is representative of pre-established PCT devices [7,16,18,20,25,26,32,33,34], whereas tri-cultures reflect the combination of the human glomerulus and PCT. While 90.0% of the fluid was not recycled into the bloodstream as shown in the simulation, approximately 36.0% was outputted into the waste collection and 64.0% was recycled into the MPS.

### 3.3. Recapitulation of Glomerulus and Proximal Tubule Functions

**Figure 5:**
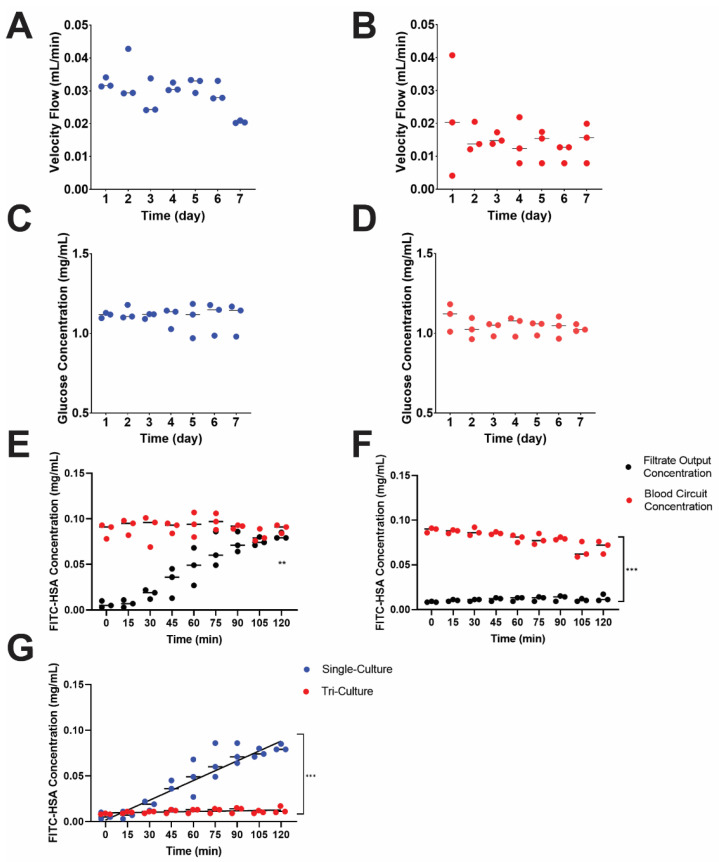
Functional comparison of glomerular and proximal convoluted tubule (PCT) MPS in single- and tri-culture conditions (*n* = 3 microsphyiological system [MPS] devices/condition). (**A**,**C**,**E**) represent single-culture experimental tests with no glomerular cells, and (**B**,**D**,**F**) depict a tri-culture MPS with a glomerulus housing unit. (**A**) Filtrate output formation in single culture. (**B**) Filtrate output formation in tri-culture. (**C**) Glucose secretion in single culture. (**D**) Glucose secretion in tri-culture. (**E**) Protein filtration of FITC-human serum albumin (HSA) in single culture. (** Comparison of individual datapoints within filtrate output, *p* < 0.01, non-parametric one-way ANOVA.) (**F**) Protein filtration of FITC-HSA in tri-culture. (*** Comparison of individual datapoints within blood circuit and filtrate output, *p* < 0.001, non-parametric one-way ANOVA and Mann–Whitney test.) (**G**) HSA concentration of filtrate output in single and tri-culture. (*** Comparison of HSA output rates in filtrate between single and tri-cultures, *p* < 0.001, linear regression, R_single_^2^ = 0.86, R_tri_^2^ = 0.20. y_tri_ = 7.2 × 10^−4^ t + 1.7 × 10^−3^ and y_single_= 2.6 × 10^−5^ t + 9.5 × 10^−3^).

Key characteristics of a functional nephron are its blood serum protein filtration, glucose resorption, and filtrate formation capacity. Two culture conditions were established to demonstrate system characteristic differences, which was denoted as a single (proximal tubule cells only, *n* = 3) and tri-culture (all cell types, *n* = 3). Throughout the seven-day operation, primary filtrate formation was collected every 24 h in conical tubes and immediately stored at −20 °C before additional assessment. Volumes of filtrate for each culture type were weighed to measure velocity flow rate (mL/min) (Figure 5A,B). The primary filtrate formation in the single culture maintained a median of approximately 30 μL/min, and the tri-culture 15.0 μL/min. The introduction of the glomerular cells and endothelial cells created a barrier effect, reducing the flow rate within the apical PCT of the MPS to decrease shear stress from 0.3 to 0.2 dyne-s/cm^2^. Results of the simulated, experimental, single, and tri-culture filtrate output and shear stress across the apical region of the PCT were shown (Figure 4C,D). While the simulated model exhibited a linear behavior based on its regression (R^2^ = 1.0), the shear stress under experimental conditions significantly decreased in the presence of cells, suggesting that simulations provided only ideal conditions and required further parametrizations.

Since the proximal tubule is responsible for nearly 90.0% of glucose reabsorption in the kidney, glucose reabsorption (mg/mL) in both single- and tri-culture devices were evaluated using the Infinity Glucose Assay (Figure 5C,D). Filtrate output volumes were sampled three times into a 96-well plate and compared to a 1 mg/mL glucose solution diluted with 0.1% benzoic acid standard. Samples were read with the plate reader at an absorbance of 340 nm. Both culture conditions lacked statistically significant differences due to the large volume of daily filtrate output (~30–40 mL/day), although there was a downward trend in the amount of glucose present in the filtrate output over the course of the 7 day culture. No significant change in both velocity (Figure 5B) demonstrated that the MPS was stable during the seven days.

On day 7 of MPS operation, 0.1 mg/mL of fluorescently tagged human serum albumin (FITC-HSA, Abcam #ab8030) diluted with 1X PBS was circulated into the MPS to assess glomerulus function and was analyzed for the amount of serum proteins in both the filtrate outlet and blood stream circuit reservoir [35]. Samples were drawn from the filtrate outlet and blood stream circuit reservoirs every 15 min for 2 h and placed in a black, 96-well plate. Samples were analyzed for fluorescence with a Synergy 2 plate reader against a standard curve (Figure 5E–G). Protein concentration in the bloodstream minimally declined from 0.1 mg/mL of FITC-HSA for both case conditions. In the tri-culture conditions, there was a statistically significant difference (*p* < 0.001) between filtrate output and bloodstream circuit output (Figure 5F). FITC-HSA concentration in tri-culture in the blood circuit output (0.083 mg/mL) was 8-fold greater than the filtrate output (0.011 mg/mL), suggesting that there is a functional filtration process in the tri-culture MPS. This behavior was not exhibited in the single-culture conditions. Single-cell culture exhibited an increase in protein concentrations in the filtrate output, while the tri-culture devices had little protein reach the filtrate output (Figure 5G). Absence of protein in the waste or filtrate stream suggests that the glomerular cells prevent the FITC-HSA from passing through the glomerular membrane, similar to the function of the glomerulus in vivo.

**Figure 6:**
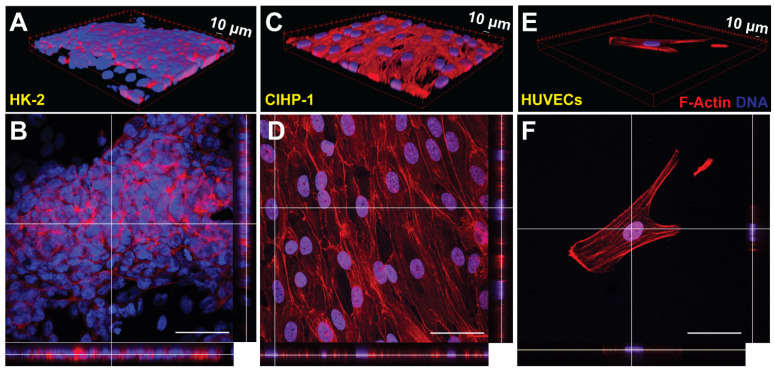
Post-experiment fluorescent-stained cell images (*n* = 9). (**A**) 3D projection of HK-2 on polycarbonate membrane. (**B**) 2D orthogonal view of HK-2 on polycarbonate membrane. (**C**) 3D projection of CIHP-1 on PES membrane. (**D**) 2D orthogonal view of CIHP-1 on PES membrane. (**E**) 3D projection of HUVECs on PES membrane. (**F**) 2D orthogonal view of HUVECs on PES membrane. Red = Phalloidin 568, blue = Hoescht 33342, and green = FITC-HSA.

Fluorescent stains provided insight into the cellular phenotype following dynamic culture. The membranes, PCT (polycarbonate) and glomerular (PES) membranes from the MPS were removed, fixed with 4% paraformaldehyde (PFA), stained with phallodin 568) and Hoescht 33342, and imaged using a Zeiss confocal microscope at 40× magnification. F-actin cytoskeleton restructuring was imaged with phallodin 568, whereas DNA in the nuclei was counterstained with Hoescht 33342. To determine the cellular confluence, qualitative 2D orthogonal views and three-dimensional (3D) views (Figure 6A–F) were obtained. Qualitatively, the HK-2 cells on the PCT membrane (Figure 6A,B) demonstrated a monolayer behavior as previously shown [28], and quantitatively represented by the 75.3% confluence. From the 3D view of Figure 6C, finger-like projections of the podocytes validated full differentiation (day 14). The endothelial cells were highly directional in the presence of flow (Figure S1), but attachment was limited by day 7 (Figure 6E,F). This may be related to HUVECs poor ability to adapt to the dynamic crossflow culture conditions. Static conditions for all cell types were imaged for comparison (Figure S2).

## 4. Discussion

Previous renal MPS models have targeted specific structures of the nephron, but regulation of xenobiotics and substrates in the blood is a concerted action of both filtration in the glomeruli and active reabsorption by the proximal tubule. The challenge in establishing an effective nephron MPS originates from fulfilling all required characteristics of kidney MPS: incorporation of renal cellular models (tubular structures or in vitro barrier models) [7,8,19,20,25,26], functional (filtration, absorption, and secretion) processes, and drug toxicity/recovery testing. In this study, the glomerulus and PCT MPS addresses most of the features of a renal MPS, while also maintaining in vivo shear stress across the apical PCT (0.4–1.5 dyne-s/cm^2^) and recirculating 64.0% of flow into the bloodstream and 36.0% into the filtrate output. Obtaining this flow split required careful design of the device based on fluid dynamic simulations and control of pressure. The tri-culture system of CIHP-1, HUVECs and HK-2 cells provides a prototype of a potentially realistic approach by recapitulating the critical functions in both the glomerulus and PCT for long-term operation. This approach overcomes some of the key problems of existing glomerulus-on-a-chip and PCT-on-a-chip designs and assessing for drug toxicity.

Geometrically, many vital attributes contribute to the innovation of the glomerulus and PCT MPS that have never been modeled previously in vitro. The MPS presents a double in vitro barrier model in both the glomerular filtration chamber and the PCT reabsorption device. Within the glomerular unit, the 30 nm pore size PES membrane was selected based on the physiological size-exclusive filtration commonly found on the human glomerular capillary walls [11,28]. The PCT barrier model highlights a multi-layered fluidic platform separated by a porous membrane, creating a dual channel system (apical and basolateral). Proximal tubule cells on the membrane selectively resorb solutes from the apical filtrate formation line to the basolateral bloodstream, reenacting the renal active and passive transport processes. This recirculation of flow was established through the T-junction component, crucial in bloodstream circuit reservoir to simulate the removal of compounds that have been reabsorbed by the renal tubule. Additionally, the PCT device was constructed from polycarbonate instead of the traditional polydimethylsiloxane (PDMS). The use of the polycarbonate PCT removes potentially unrealistic absorption of chemicals in the PDMS and that the devices are highly robust, autoclavable, and reused up to 10 times. The MPS design fosters low-cost manufacturing and minimizes wasted resources from single-use devices. Furthermore, an optimal common cell culture medium (ESFM) was determined based on a series of viability and proliferation testing in all three cell types without animal products or antibiotics (fetal bovine serum or penicillin). The lack of animal-based products furthers the accuracy and applicability of the MPS to human organ functionality. Throughout the in vitro experiments, comparisons of the single- and tri-culture systems determined the significance of modeling concerted structures of the nephron as evidence by the HSA filtration in the tri-culture in comparison to the single culture). Results from velocity flow rate and protein concentration measurements validate how the glomerulus and PCT MPS greatly impact the retention of functional cell–cell and cell–tissue interactions.

Throughout the MPS experiments, comparisons of the single- and tri-culture systems determined the significance of modeling concerted structures of the nephron. The single culture, containing only PCT cells, is illustrative of the conventional PCT devices [11,18,20,26,32] and cannot filter serum albumin. The tri-culture, containing podocytes, endothelial cells, and proximal tubule cells, features filtration attributes that single cultures lack. Since filtration occurs in the glomerular filtration barrier mainly controlled by the glomerular and endothelial cells [36], co-culturing these cell types is anatomically crucial, but has previously proved challenging in culturing on a single porous membrane. Because both cell types are highly differentiated, specialized, and interdependent cell types [37,38], selection of the cells in the model is vital in evaluating the MPS. CIHP-1 cells, representative of the podocytes, were selected based on its immortalized, homogenous characteristics. To ensure that the MPS establishes efficacy, homogeneity minimizes outliers and inconsistencies. Similarly, HUVECs were selected based on prevalent use in endothelial cell studies [39,40] and derivation from humans. Reabsorption is mostly contributed by the proximal tubule cells as modeled by the HK-2 cells, which were selected based on extensive use previous work [8,11,20,28]. From the comparative results, the single and tri-culture were indicative of the significance of establishing a multi-cellular model in future kidney research. The differences in velocity flow rate and protein concentrations validate how the glomerulus and PCT MPS greatly impact the retention of functional cell–cell and cell–tissue interactions.

To achieve animal-by-product-free culture conditions, multiple media types were tested. Based on the results of the viability and proliferation testing (Figure S3), all three cell types were adapted to an optimal common cell culture medium (ESFM) prior to dynamic experimentation. For the glomerular functional assay, human serum albumin was used (FITC-HSA, 67.0 kDa). Cells within the glomerulus and PCT MPS demonstrated functional activity through the retention of HSA (~67 kDa) from circulating in the bloodstream pathway. The protein concentrations were depicted in both the plate reader fluorescence readings, but also in the confocal images, where there was an uptake of the green fluorescent protein in the podocytes (Figure S4). Previous works [18,20] found that dynamic conditions results in cytoskeletal reorganization and junctional reformation in PCT-derived cells. These outcomes were not clearly observed in the glomerulus and PCT MPS due to the low shear stress to exhibit healthy renal conditions and longer culture time. Although the cells in this prototype model did not achieve confluency, functional activity was demonstrated through the retention of HSA (~67 kDa) from circulating in the bloodstream pathway, while secreting concentrations of glucose from the filtrate output. Importantly, these features were not observed in the single culture (PCT only cells) demonstrating the potential usefulness of the design, which would be further enhanced by use of primary cells or other extracellular matrices (collagen IV and laminin) [7,26,36,41,42,43,44]. Overall, the glomerulus and PCT MPS design offers a potentially effective tool for assessing preclinical drug adsorption, distribution, metabolism, elimination, and toxicity (ADMET) testing, modeling disease for drug treatment regime trials, and understanding cellular crosstalk.

## 5. Conclusions

The glomerulus and proximal tubule microphysiological system presented in this study successfully recapitulates kidney filtration and reabsorption properties. A 2D computational model of the velocity and shear rate profiles provided a template for achieving a shear stress of 0.4–1.5 dynes/cm^2^ within the device. Single- and tri-cellular in vitro models were characteristically different in their functional capabilities. Though both systems operate at 0.7 dynes/cm^2^ for 7 days, there are clear differences in the filtrate output. The significance of the tri-culture is verified by the glomerular cells filtering human serum albumin, acting as filtration barrier both biologically and mechanically. In this work, the tri-culture presents a more realistic cellular model of the glomerulus and PCT.

## Figures and Tables

**Table 1:** MPS Critical Parameterizations.

Inlet Mass Flow Rate (10^−7^ kg/s)	Tubing ID Diameter (mm)	Filtrate Output Length (mm)	Bloodstream Output Length (mm)
2.66	0.25	457	622
5.15		474	660
6.67	0.51	491	698
7.55		508	736
10.1		525	774

## Data Availability

The data that support the findings of this study are available from the corresponding author upon reasonable request.

## Supplementary Materials

The following are available online at https://www.mdpi.com/article/10.3390/mi12080983/s1, Figure S1: Directionality measurements of tri-culture cells; Figure S2: 2D confocal images of cells grown in ESFM under static conditions for 7 days; Figure S3: Growth curve for renal cells in four different types of medium; Figure S4: Localization of FITC HSA in CIHP-1.
