# Supplementary material for: Modelling Renal Filtration and Reabsorption Processes in a Human Glomerulus and Proximal Tubule Microphysiological System
Source: Micromachines (Basel). 2021 Aug 19;12(8):983. doi: 10.3390/mi12080983 (PMC8398588; doi:10.3390/mi12080983)
Supplement: Supplementary file 1 [file micromachines-12-00983-s001.zip › micromachines-1329336-supplementary.pdf]

micromachines

Supplementary Material

Modelling Renal Filtration and Reabsorption Processes in a Human Glomerulus and Proximal Tubule Microphysiological System

Stephanie Y. Zhang and Gretchen J. Mahler *

Department of Biomedical Engineering, The State University of New York at Binghamton, Binghamton, NY 13902, USA; szhan152@binghamton.edu
* Correspondence: gmahler@binghamton.edu

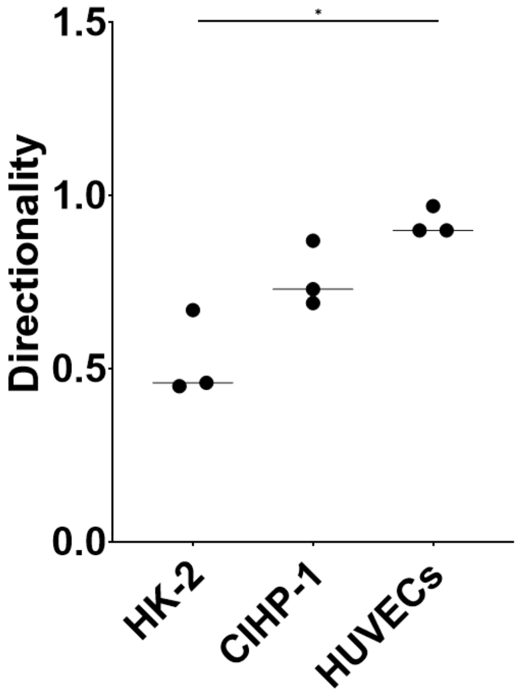

Figure S1. Directionality measurements for tri-culture cells (HK-2, CIHP-1, HUVECs). (* Comparison of individual datapoints within each cell type, $p<0.05$, non-parametric one-way ANOVA).

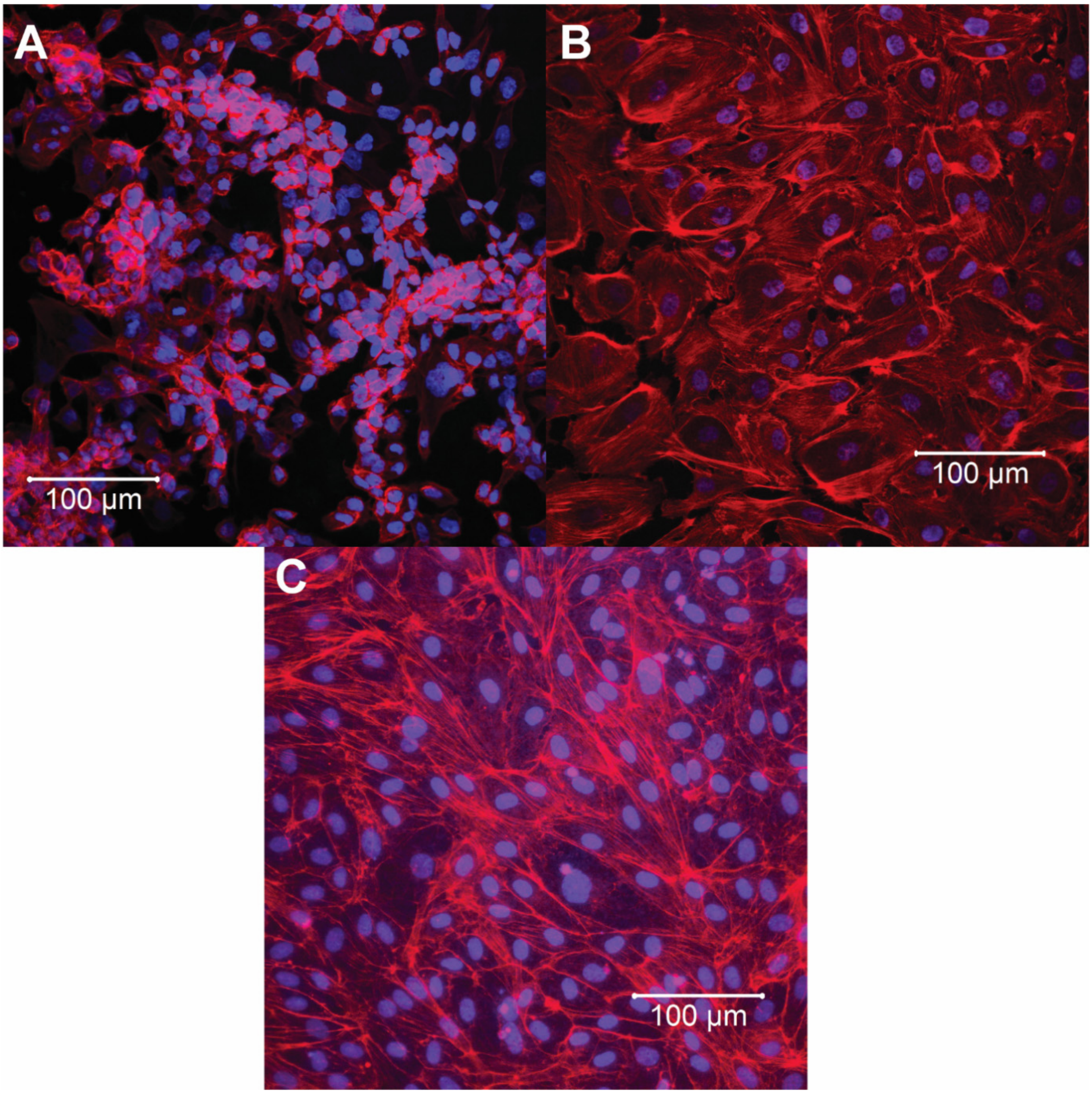

Figure S2. 2D confocal images of cells grown in ESFM under static conditions for 7 days. (**A**) HK-2 cells on polycarbonate membrane. (**B**) CIHP-1 cells on PES membrane. (**C**) HUVECs on PES membrane. Red= F-actin, Blue= DNA. Scale bar = 100 µm.

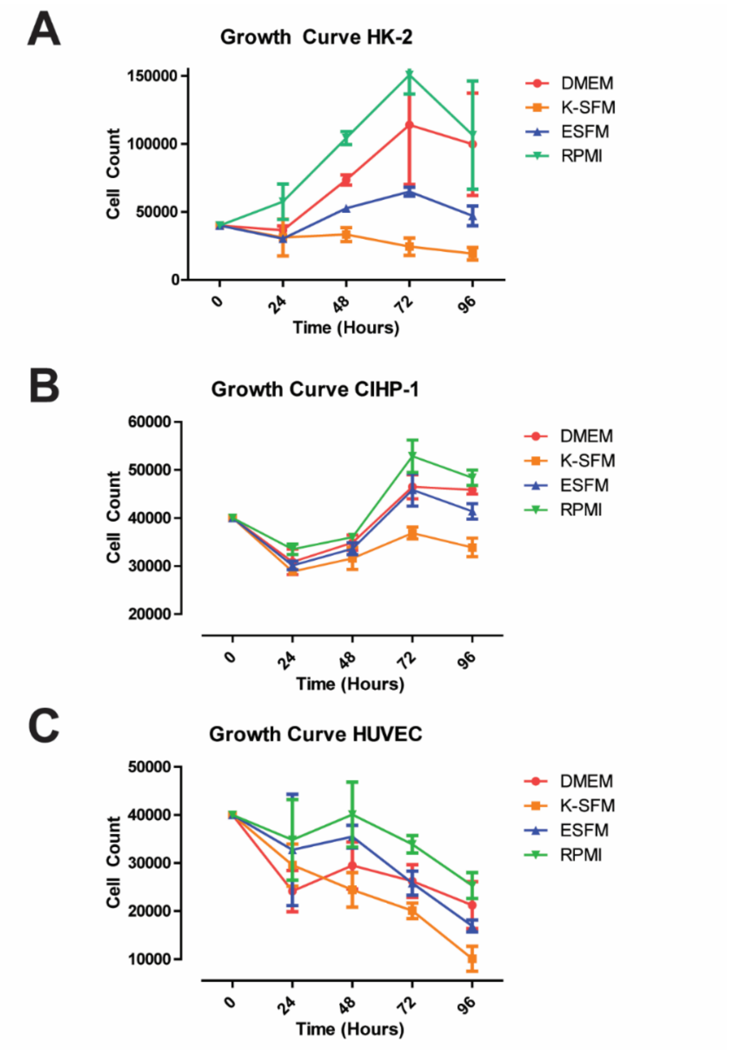

Figure S3. Growth curve for renal cells in four different types of medium. The four different types of medium were Dulbecco's Modified Eagle Medium (DMEM), Kerotinocyte-Serum Free Media (KSFM), Endothelial Serum Free Medium (ESFM), and Roswell Park Memorial Institute media (RPMI-1640) and results are shown for the static culture of (**A**) Proximal tubule (HK-2) cells, (**B**) Conditionally immortalized human podoctyes (CIHP-1) cells, and (**C**) Human umbilical vein endothelial cells (HUVECs) for 96 hours.

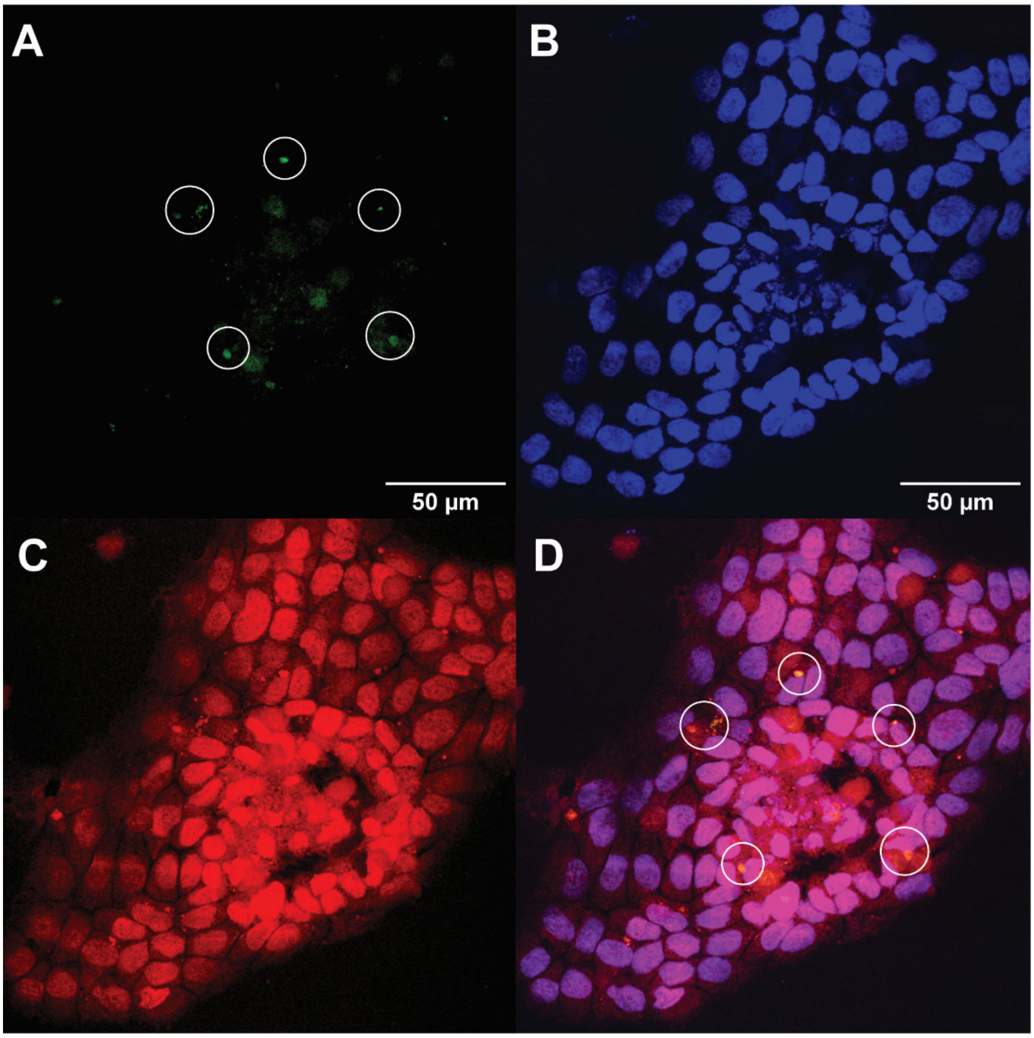

Figure S4. Localization of FITC HSA in CIHP-1. 2D confocal image at 40x magnification were taken of (**A**) FITC-HSA (green, circled) within the cell monolayer, (**B**) DNA (blue, Hoescht 33342), and (**C**) F-actin (red, Phalloidin 568). (**D**) Merged channels. Scale bar = 50 µm.
